# Short-term outcomes after simultaneous gastrectomy plus cholecystectomy in gastric cancer: A pooling up analysis

**DOI:** 10.1515/med-2022-0605

**Published:** 2023-02-06

**Authors:** Bing Kang, Xu-Rui Liu, Dong Peng

**Affiliations:** Department of Gastrointestinal Surgery, The First Affiliated Hospital of Chongqing Medical University, Chongqing, 400016, China; Department of Clinical Nutrition, The First Affiliated Hospital of Chongqing Medical University, Chongqing, China

**Keywords:** gastric cancer, gastrectomy, cholecystectomy, short-term outcomes, meta-analysis

## Abstract

The purpose of this study was to evaluate the short-term outcomes after simultaneous gastrectomy plus cholecystectomy in gastric cancer patients. PUBMED, EMBASE, and the Cochrane Library were searched from inception to Apr 15, 2021. Short-term surgical outcomes were compared between the simultaneous gastrectomy plus cholecystectomy group and the gastrectomy only group. Five retrospective studies with 3,315 patients and 1 randomized controlled trial with 130 patients were included. There was no significant difference in age, sex, surgical methods, or reconstruction. In terms of short-term outcomes, no significance was found in postoperative complications (odds ratio, OR = 1.08, *I* ^2^ = 24%, 95% CI = 0.78–1.50, *P* = 0.65), postoperative biliary complications (OR = 0.98, *I* ^2^ = 0%, 95% CI = 0.43–2.25, *P* = 0.96), mortality (OR = 1.28, *I* ^2^ = 0%, 95% CI = 0.49–3.37, *P* = 0.61), and postoperative hospital stay (MD = −0.10, *I* ^2^ = 0%, 95% CI = −0.73–0.54, *P* = 0.77) between the two groups. Simultaneous gastrectomy plus cholecystectomy in gastric cancer patients is safe and does not increase the short-term outcomes.

## 1 Introduction

Gastric cancer is the fifth most diagnosed cancers worldwide and the third most common cause of cancer-related deaths [1,2]. Surgery is still a mandatory backstone in the treatment for gastric cancer patients. The main procedures are gastrectomy plus D2 lymph node dissection [2].

Many studies have reported that the incidence of cholecystolithiasis after gastric cancer surgery was higher than that of the general population, and some of which required a secondary surgery [3,4,5,6,7,8]. It might be associated with the changes in the vagus nerve branches after surgical dissection and the changes in the gastrointestinal anatomy after reconstruction [9,10,11]. Considering the high incidence of cholecystolithiasis after gastrectomy, simultaneous cholecystectomy was not time-consuming, and there was basically little increased risk to the patients; therefore, some authors proposed simultaneous gastrectomy plus cholecystectomy [12,13].

However, whether cholecystectomy should be performed at the same time with gastrectomy remains controversial. The Cholegas study reported that combined cholecystectomy during gastrectomy did not increase the risk of perioperative morbidity and mortality [14]. But another study reported that simultaneous gastrectomy plus cholecystectomy increased the risk of perioperative morbidity [15]. The purpose of this study was to evaluate the surgical mortality and morbidity after combined gastrectomy plus cholecystectomy in gastric cancer patients.

## 2 Materials and methods

### 2.1 Structure of meta-analysis

The current study conformed to the Preferred Reporting Items for Systematic Reviews and Meta Analyses (PRISMA) statement stringently [16]. The registration ID of this meta-analysis on PROSPERO is CRD42021252274, and the link is https://www.crd.york.ac.uk/prospero/display_record.php?ID=CRD42021252274.

### 2.2 Literature search

Two authors searched through PUBMED, EMBASE, and the Cochrane Library for literature independently, and the deadline was Apr 15, 2021. The strategies were as follows: (“gastric tumor” OR “gastric cancer”) AND (“cholecystectomy” OR “gall stone” OR “cholecystolithiasis” OR “cholelithiasis”). The language was restricted to publications in English.

The inclusion criteria of this current study were as follows: (1) studies comparing surgical endpoints between simultaneous gastrectomy plus cholecystectomy group and gastrectomy only group in gastric cancer patients; (2) at least one short-term outcome was reported in the included studies, the short-term outcomes were as follows: postoperative overall complications, postoperative biliary complications, postoperative mortality, or postoperative hospital stay. The exclusion criteria were as follows: (1) reviews, conference, comments, case reports, and/or non-original articles and (2) studies with none of these short-term complications or endpoints. Group discussions were conducted on disputed points about inclusion.

### 2.3 Data extraction and quality assessment

Two authors searched the literature independently. The titles and abstracts were screened for the relevant content, then we checked full texts carefully according to the inclusion and exclusion criteria. A group discussion would be held on disagreement, and if there was still a dispute, a third author would be involved in the final decision.

For the included studies, two authors extracted data independently. The data extracted were as follows: first author, publication year, study period, country, study type, sample size, postoperative overall complications, postoperative biliary complications, postoperative hospital stay, and postoperative mortality. The unclear data were evaluated through emails to the original authors if necessary. Discussion in groups if disagreements appeared.

The main outcome of this study was the postoperative overall complications. Secondary outcome included postoperative biliary complications, mortality rate, and postoperative hospital stay.

We used the risk of bias in non-randomized studies of interventions (ROBINS-I) tool to assess the quality of included studies, and each study was evaluated in three domains including selection, comparability, and results [17]. The senior author completed the assessment independently.

### 2.4 Statistical analysis

In the study, the odds ratio (OR) of dichotomous variables and mean difference (MD of continuous variables were calculated, and 95% confidence interval (95% CI) was calculated, respectively. The statistical heterogeneity of the included studies was assessed by using the *I* ^2^ value. When *I* ^2^ > 50%, the random-effect model was used, and high heterogeneity was considered, while *P* < 0.1 was considered statistically significant. Otherwise, we used the fixed effects model and statistically significant was defined as *p* < 0.05 [18]. We used RevMan 5.3 (The Cochrane Collaboration, London, United Kingdom) for data analysis in this study.

## 3 Results

### 3.1 Study and patient characteristic

**Figure 1:**
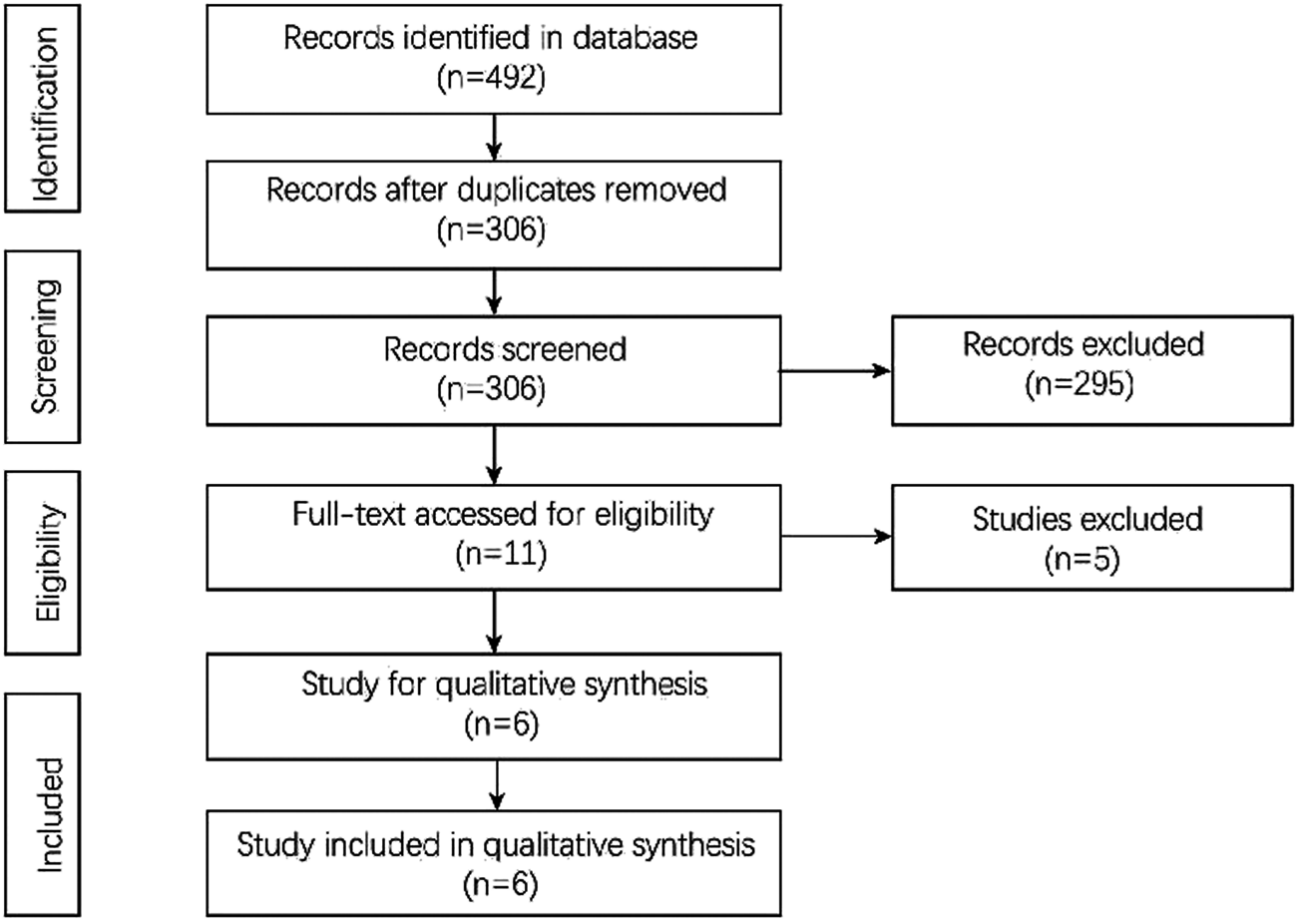
Flowchart of study selection.

492 studies were identified in the databases, of which 186 studies were removed for duplication. 295 studies were removed after screening the titles and abstracts and 11 studies were evaluated for full text review. Finally, six studies [14,15,19,20,21,22] which compared short-term outcomes after simultaneous gastrectomy plus cholecystectomy and only gastrectomy in gastric cancer patients were included in this study. The flow chart is shown in Figure 1.

There were 460 patients who underwent simultaneous gastrectomy plus cholecystectomy and 2,985 patients who underwent gastrectomy only for gastric cancer in the 6 studies. One is randomized controlled trial (RCT) from Italy and the other five were retrospective studies from Korea, China, and Japan. The publication year was from 2009 to 2019, and the study date was from 1988 to 2018. The level of the ROBINS-I is shown in Table 1.

**Table 1:** Characteristics of the studies included in the meta-analysis

Author	Year published	Country	Study design	Study date	Sample size		Gender (male/female)		ROBINS-I
					Gastrectomy + cholecystectomy	Gastrectomy	Gastrectomy + cholecystectomy	Gastrectomy	
Bernini et al. [14]	2013	Italy	RCT	2008–2012	65	65	38/27	37/28	High
Jeong et al. [19]	2009	Korea	Retrospective	2003–2007	26	364	16/10	242/122	Moderate
Kim et al. [15]	2019	Korea	Retrospective	2011–2016	242	242	144/98	141/101	Moderate
Lai et al. [21]	2013	China	Retrospective	1988–1998	58	387	39/19	234/153	Moderate
Tan et al. [22]	2019	China	Retrospective	2010–2018	62	1,691	Unknown	Unknown	High
Woo et al. [20]	2012	Japan	Retrospective	2005–2009	7	236	4/3	126/110	Low

RCT, randomized controlled trial; ROBINS-I, the risk of bias in non-randomized studies of interventions.

### 3.2 Quantitative data synthesis

#### 3.2.1 Baseline information

Between the Gastrectomy + Cholecystectomy group and the Gastrectomy only group, the results showed that no significant difference was found in age (MD = 1.40, *I* ^2^ = 0%, 95% CI = 0.50–3.30, *P* = 0.15) [15,18] or males (MD = 1.08, *I* ^2^ = 0%, 95% CI = 0.83–1.40, *P* = 0.58) [14,15,18,19,20]. The total gastrectomy (MD = 0.92, *I* ^2^ = 0%, 95% CI = 0.64–1.32, *P* = 0.64) [14,15,18,19] and reconstruction method (MD = 0.89, *I* ^2^ = 0%, 95% CI = 0.62–1.28, *P* = 0.53) [14,15,18,19] had no significant difference (Table 2).

**Table 2:** Summary of characteristics between gastrectomy plus cholecystectomy group and gastrectomy group

Characteristics	Studies	Participants (gastrectomy + cholecystectomy/gastrectomy)	MD/OR (95% CI)	Heterogeneity
Baseline information				
Age	2	268/606	1.40 [0.50, 3.30]; *P* = 0.15	*I* ^2^ = 0%; *P* = 1.00
Male	5	398/1,294	1.08 [0.83, 1.40]; *P* = 0.58	*I* ^2^ = 0%; *P* = 0.90
Surgical methods and reconstruction				
Total gastrectomy	4	340/907	0.92 [0.64, 1.32]; *P* = 0.64	*I* ^2^ = 0%; *P* = 0.58
Reconstruction	3	340/907	0.89 [0.62, 1.28]; *P* = 0.53	*I* ^2^ = 0%; *P* = 0.42
Postoperative hospital stay (days)	2	268/606	−0.10 [−0.73, 0.54]; *P* = 0.77	*I* ^2^ = 0%; *P* = 0.39

CI, confidence interval.

#### 3.2.2 Primary outcomes

**Figure 2:**
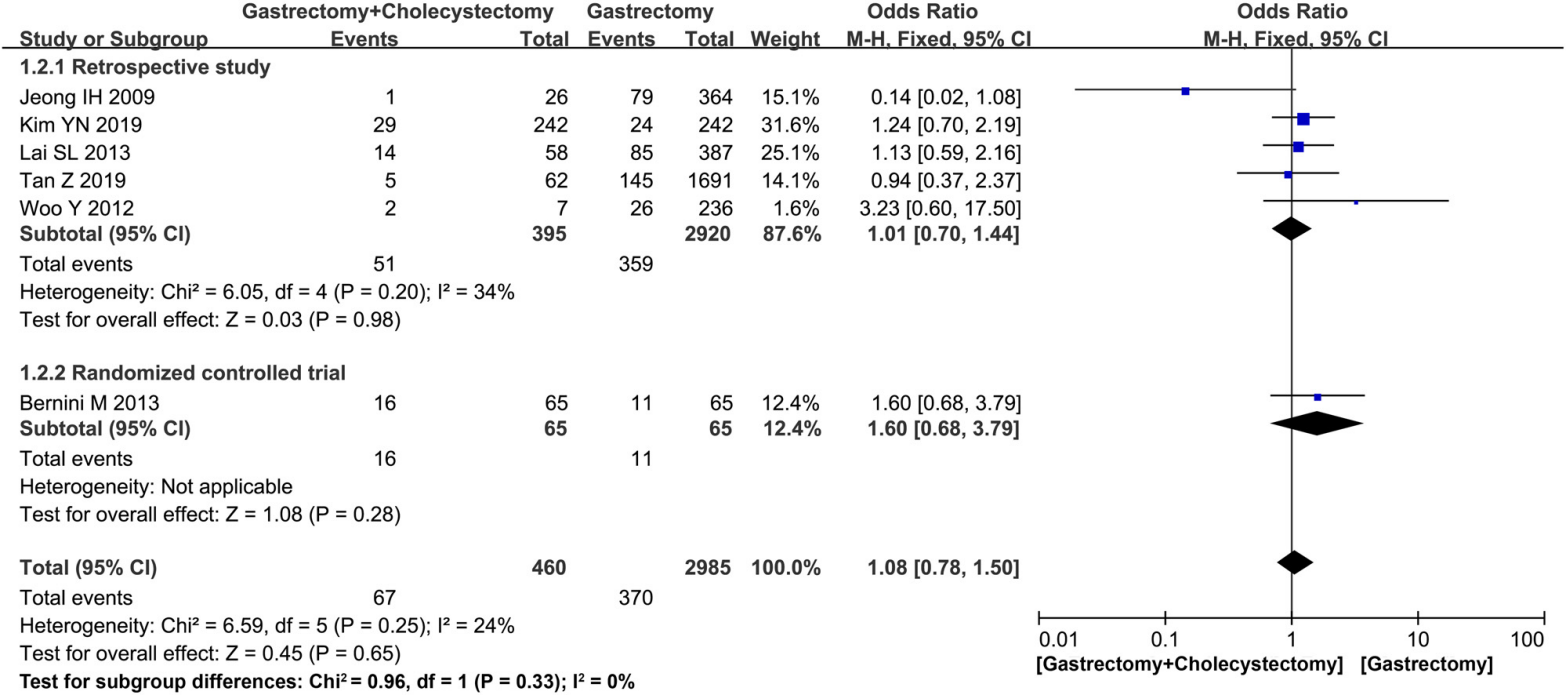
Forest plot showing postoperative overall complications.

Postoperative complications were divided into two groups including postoperative overall complications and postoperative biliary complications. All six studies [14,15,19,20,21,22] reported the overall postoperative complications, the one RCT [14] did not find any significant difference between the two groups (MD = 1.60, 95% CI = 0.68–3.79, *P* = 0.28). Even for the five retrospective studies [15,19,20,21,22], there was no significant difference between the two groups (MD = 1.01, *I* ^2^ = 34%, 95% CI = 0.70–1.44, *P* = 0.98) (Figure 2).

#### 3.2.3 Secondary outcomes

**Figure 3:**
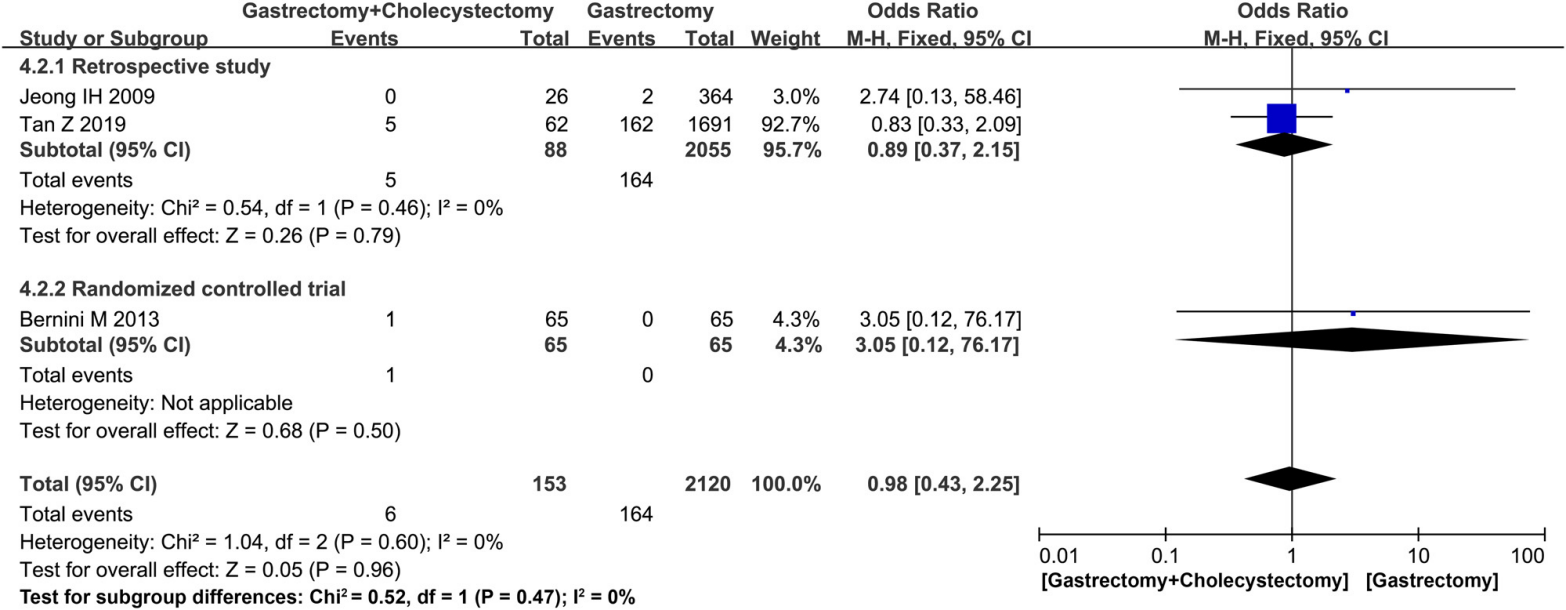
Forest plot showing postoperative biliary complications.

**Figure 4:**
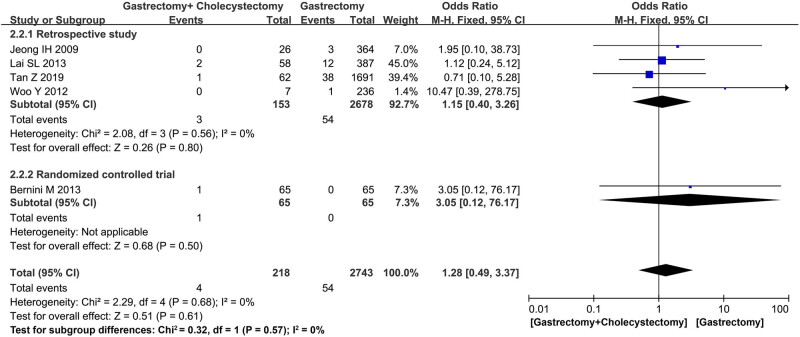
Forest plot showing postoperative mortality.

Three studies [14,19,22] reported the postoperative biliary complications, the one RCT [14] did not find any significant difference between the two groups (MD = 3.05, 95% CI = 0.12–76.17, *P* = 0.50), and the analysis of the other two retrospective studies [19,22] also reported that there was no significance in the two groups (MD = 0.89, *I* ^2^ = 0%, 95% CI = 0.37–2.15, *P* = 0.79) (Figure 3). Five studies [14,19,20,21,22] including 2,961 patients reported the short-term mortality. No significant difference was found between the two groups (MD = 3.05, 95% CI = 0.12–76.17, *P* = 0.50) in the one RCT [14], and also in the analysis of four retrospective studies [19,20,21,22] (MD = 1.15, *I* ^2^ = 0%, 95% CI = 0.40–3.26, *P* = 0.80) (Figure 4). Two studies reported the postoperative hospital stay, and no significance was found (MD = −0.10, *I* ^2^ = 0%, 95% CI = −0.73–0.54, *P* = 0.77) (Table 2).

### 3.3 Sensitivity and publication bias

**Figure 5:**
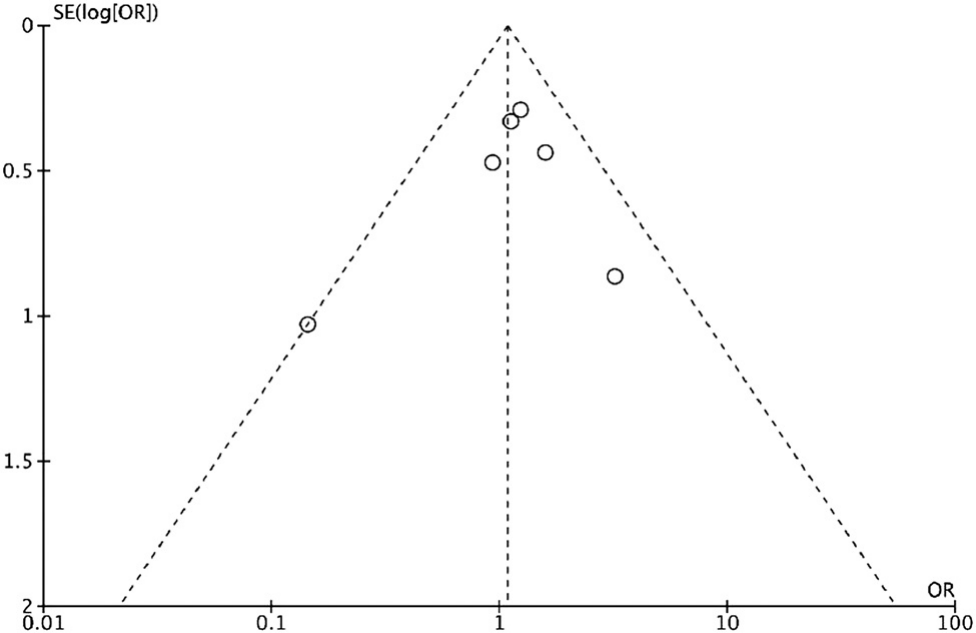
Funnel plot of postoperative overall complications in the included studies showing no evidence of publication bias. SE: standard error, OR: odds ratio.

Sensitivity analyses were performed to assess the robustness of the results by repeating analyses of removing one study at a time, and every result did not change effect size or overall heterogeneity. The results showed no publication bias in the funnel plots (Figure 5).

## 4 Discussion

Gastrectomy is the main treatment method of gastric cancer [1,23]. The safety and feasibility of simultaneous gastrectomy plus cholecystectomy are still in debate [24,25]. In this study, 6 studies with 3,445 patients were included. For baseline information and surgical methods, the simultaneous gastrectomy plus cholecystectomy group and gastrectomy only group had no significant difference in age, sex, total gastrectomy, or reconstruction method. No significance was found in postoperative complications including overall complications, biliary complications, mortality, and postoperative hospital stay between the two groups.

Many studies have reported a 3–4-fold increase in the incidence of gallstones 5 years after gastric surgery, approximately 6% of patients undergoing upper gastrointestinal tract surgery would probably require cholecystectomy during follow-up [14,26,27]. There are two mechanisms for this phenomenon: First, after the surgical dissection of the vagus nerve branches in the gastrectomy, it might play an important role in the change in plasma cholecystokinin concentrations and gallbladder emptying, resulting in stone formation [28,29,30]. Second, reconstruction such as Billroth II and Roux-en-Y excluded food passage through the duodenum, which might lead to higher risk of gallstone formation [31,32,33].

Considering the safety of the operation and postoperative complications, it was controversial whether gastrectomy plus cholecystectomy were performed simultaneously in gastric cancer patients. Previous study reported that the patients with gastric cancer undergoing the gastrectomy plus cholecystectomy simultaneously had increased risk of perioperative morbidity [15]. However, another two studies reported that simultaneous gastrectomy plus cholecystectomy did not influence the mortality [34,35]. In our study, gastric cancer patients in the simultaneous gastrectomy plus cholecystectomy group had a 28% increase in mortality over the gastrectomy group, although the differences were not significant. Meanwhile, we found no significant difference in postoperative overall complications and biliary complications in this study. On the other hand, simultaneous gastrectomy plus cholecystectomy in gastric cancer patients did not increase the postoperative hospital stay than gastrectomy group.

Nevertheless, the sample size of studies about the duration of surgery and the intraoperative blood loss was limited. Only one study showed the operative time was significantly shorter in the gastrectomy only group than in the simultaneous cholecystectomy group [15]. However, the other four studies presented a difference between the two groups [14,19,20,21]. About the intraoperative blood loss, the same as duration of surgery, Kim mentioned that the simultaneous cholecystectomy group showed more intraoperative blood loss than the gastrectomy only group [15]. But no significant difference was found in the other two studies [14,20]. Therefore, more large samples and prospective studies were needed in the future.

Although implementing gastrectomy in gastric cancer patients might increase the risk of cholecystolithiasis, but not all patients would have cholelithiasis after gastrectomy and would need subsequent cholecystectomy [3,5,36,37]. A minimally invasive cholecystectomy was feasible even in those patients who underwent gastric surgery previously [38,39,40]. Although there is still controversy in the duration of surgery and intraoperative blood loss between the two groups, it did not affect the incidence of postoperative overall complications, biliary complications, mortality, and postoperative hospital stay. Overall, we suggest combined gastrectomy plus cholecystectomy is safe and does not change short-term outcomes.

There are several limitations. First, only six studies were included and only one was RCT, while five were retrospective studies. Second, there was lack of complete baseline data such as tumor depth and TNM staging. Third, the data form of duration of surgery and intraoperative blood loss were limited, which may result in heterogeneity and the results might not be precise enough. Therefore, RCTs with large samples and high quality should be carried out in the future.

## 5 Conclusion

In conclusion, simultaneous gastrectomy plus cholecystectomy is safe and does not increase the short-term outcomes in gastric cancer patients.
